# Multichannel Coupling in the Electronic Excitation of Pyrimidine Induced by Low-Energy Electron Impact

**DOI:** 10.1021/acs.jpca.5c07765

**Published:** 2026-01-23

**Authors:** Murilo O. Silva, Márcio H. F. Bettega, Romarly F. da Costa

**Affiliations:** † Instituto Federal do Paraná, Campus Avançado Goioerê, Rodovia Luiz Dechiche, no number, Goioerê, Paraná 87360-000, Brazil; ‡ Departamento de Física, Universidade Federal Do Paraná, Caixa Postal 19044, Curitiba, Paraná 81531-980, Brazil; § Centro de Ciências Naturais E Humanas, 74362 Universidade Federal Do ABC, Santo André, São Paulo 09210-580, Brazil

## Abstract

We present elastic and electronically inelastic cross sections for the scattering of low-energy electrons by pyrimidine. The calculations employed the Schwinger multichannel method for impact energies up to 50 eV. The cross sections were computed within the minimal orbital basis for single configuration interactions (MOB-SCI) strategy, considering from 1 to 295 open channels. Our results are compared with theoretical and experimental data available in the literature. Although we found good agreement in the elastic scattering, there are discrepancies between the inelastic cross sections, which are discussed in light of the multichannel coupling. We also estimated the total cross section by summing the elastic, electronically inelastic, and total ionization cross sections, where the latter was obtained using the binary-encounter-Bethe model.

## Graphical abstract

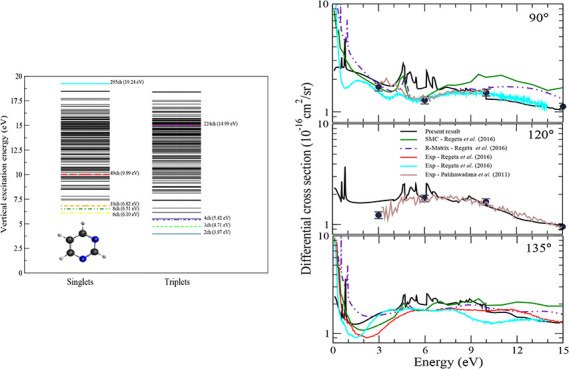

## 1 Introduction

Low-energy electrons (LEEs) play a central role in radiation chemistry and in the understanding of the biological effects caused by ionizing radiation. They are generated in large quantities when high-energy radiation, such as X-rays, γ-rays, and alpha or beta particles, interacts with matter, producing secondary electrons that trigger a series of chemical reactions. LEEs with energies less than 10 eV, in particular, can interact resonantly with molecules, giving rise to radicals and other reactive species that subsequently lead to chemical transformations and cell damage.^1,2^ Owing to their moderate energy, LEEs have a high probability of interaction, making them responsible for a significant portion of the damage effects in the cellular environment due to radiation. The relevance of these electrons extends from fundamental scientific understanding on DNA strand breakage to practical applications in medicine, specifically in the development of more refined protocols to be used in cancer treatment. In fact, accurate electron scattering cross sections are crucial for modeling radiation damage in biological systems, as they are used in Monte Carlo simulations to track electron trajectories and predict how radiation interacts with matter.^3,4^ Therefore, a detailed investigation of LEEs behavior and their chemical interactions within living cells is essential for advancing fields that involve the use of radiation so as to further assess the extent of its biological impacts.^5,6^

Motivated by this context, our study focuses on the pyrimidine (C_4_H_4_N_2_) molecule, which is an organic heterocyclic compound that has a structure similar to benzene, but includes two nitrogen atoms in its ring, as illustrated in Figure 1 (generated with MacMolPlt^7^). This molecule is widely used as a model system in studies of electron interactions with the DNA and RNA nitrogenous bases, since three of the main nucleotide bases—cytosine, thymine and uracil—are derivatives of pyrimidine.^8^ Furthermore, heterocyclic compounds are abundant in nature and play a very important role in life, as their structural subunits are present in many natural products, such as vitamins, antioxidant, antiviral and others.^9^ This relevance makes them a subject of great interest in the design of biologically active molecules. Within this class, nitrogen-containing heterocycles are particularly important in medicinal chemistry, significantly contributing to biology and industry, as well as aiding in the understanding of vital processes.^10^

**Figure 1:**
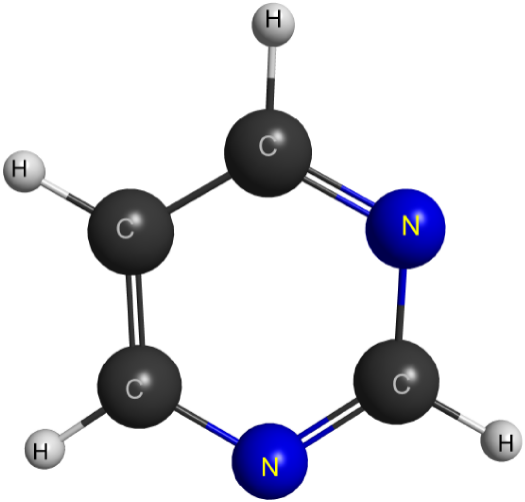
Ball and stick model of the pyrimidine molecule.

Nenner and Schulz^11^ were pioneers in studying the interaction between electrons and the pyrimidine molecule using the electron transmission spectroscopy (ETS) technique. The authors identified three resonances located at 0.22, 0.77, and 4.24 eV. The first two were characterized as purely shape resonances, while the third exhibited a mixed character, involving both shape and core-excited features. Later, Modelli et al.,^12^ also using the ETS technique, observed similar resonances at 0.39, 0.82, and 4.26 eV. Regeta et al.,^13,14^ employing an electron impact spectrometer, confirmed the presence of these three resonances, with peaks at 0.27, 0.70, and 4.35 eV and, finally, scattering calculations using the R-matrix method identified resonances at 0.53, 0.96, and 4.78 eV, consistent with previous experimental assignments.

Maljković et al.^15^ presented experimental and theoretical results of differential cross sections for energies ranging from 50 to 300 eV. The measurements were obtained using the crossed-beam technique for scattering angles between 20° and 110°, and the theoretical results were calculated using the independent atom model with a screened additivity rule correction (IAM+SCAR). Palihawadana et al.^16^ also presented experimental and theoretical results for the elastic scattering of electrons by the pyrimidine molecule, employing the Schwinger multichannel (SMC) and IAM+SCAR methods. The experimental data were obtained using a crossed electron-molecule beam spectrometer and the relative flow technique. These authors found a good agreement with the positions of the resonances previously reported in the literature, as well as a strong correspondence between theoretical and experimental results at low energies. However, they highlighted that, for energies above 10 eV, additional channels should be included in the calculations to improve the agreement. Mašín et al.^17^ conducted a combined theoretical (using the R-matrix method) and experimental (with the electron energy loss technique) study, providing both elastic and inelastic cross sections, where the authors identified the formation of resonances at 0.21, 0.63, and 5.15 eV. The experimental data for inelastic scattering were presented as energy bands due to experimental limitations. Sanz et al.^18^ presented a theoretical study that combines the R-matrix and IAM+SCAR methods, using the first to describe lower energies and the second for higher energies (up to 10 keV). Their work covers both elastic and inelastic cross sections, showing good agreement with experimental data from the literature. Baek et al.,^19,20^ using the crossed-beam technique, and Bug et al.,^21^ who developed modeled functions to calculate cross sections from a comprehensive set of available experimental data, also presented results for elastic, inelastic and total cross sections that align well with the existing literature. Zecca et al.^22^ presented results for the total cross section in electron scattering, using the IAM+SCAR method, with and without the inclusion of contributions from rotational excitations. Fuss et al.^23^ provided experimental data for the total cross section, obtained from an experiment with magnetic confinement. Levesque et al.^8^ contributed with data for the inelastic cross section at low energies and, Sinha and Antony^24^ using the spherical complex optical potential (SCOP) formalism to calculate the scattering amplitudes, reported elastic and total cross sections in the energy range from 10 eV to 5 keV, achieving good agreement with the data available in the literature. Finally, Luthra et al.^25^ presented theoretical results on electron scattering from the pyrimidine molecule in the energy range from 1 to 5 keV. The elastic cross sections were calculated using the single-center expansion (SCE) formalism with local potentials, while the total cross section was obtained by summing the elastic cross section with the ionization cross section calculated using the binary-encounter-Bethe (BEB) model. At low energies, the authors reported only a reasonable agreement with the literature, which they attributed to the fact that local potentials do not adequately describe polarization effects in this energy region. In contrast, at higher energies, the agreement with previously reported data is excellent.

In this work, we present integral and differential cross sections for elastic and electronically inelastic scattering of electrons by the pyrimidine molecule as well as total cross sections, considering impact energies of up to 50 eV. We employed the SMC method^26,27^ implemented with pseudopotentials^28^ to obtain the scattering amplitudes. To account for the effects of channel coupling (ranging from 1 to 295 open channels), we applied the minimal orbital basis for single configuration interactions (MOB-SCI) strategy.^29^ For electronically inelastic scattering processes, we compared our results with the experimental data available in the literature, where excitations are grouped into energy bands rather than individual state transitions. Accordingly, we identified the states within the experimental bands and summed their contributions for a straight comparison and more accurate analysis. To determine the total cross section, we combined the elastic and electronically inelastic contributions with the total ionization cross section, calculated using the BEB model,^30^ which is widely recognized and validated in the literature.

This article is structured as follows: Sections 2 and 3 provide a brief description of the theoretical method employed and the computational details used in the scattering calculations. Section 4 presents the results and their discussion. Finally, the conclusions are outlined in Section 5.

## 2 Theory

The elastic and electronically inelastic cross sections were obtained using the SMC method^26,27^ implemented with the norm-conserving pseudopotentials proposed by Bachelet, Hamann, and Schlüter (BHS).^31^ These pseudopotentials were used to represent the nuclei and core electrons of the heavy atoms. The SMC method is an extension of the Schwinger variational principle and incorporates essential effects that occur during the electron-molecule scattering process, such as exchange interaction, target polarization effect, and multichannel coupling. Since the SMC method has been reviewed in ref 32, here we will present only its aspects that are pertinent to the present calculations. In the SMC method, the resulting expression for the scattering amplitude is as follows:

1 $$f\left({\mathrm{k⃗}}_{f},{\mathrm{k⃗}}_{i}\right)=-\frac{1}{2\pi }\underset{m,n}{\sum }\left\langle S_{{\mathrm{k⃗}}_{f}}\right|V\left|\chi_{m}\right\rangle (d^{-1}{)}_{mn}\left\langle \chi_{n}\right|V\left|S_{{\mathrm{k⃗}}_{i}}\right\rangle$$

where

2 $$d_{mn}=\left\langle \chi_{m}\right|A^{\left(+\right)}\left|\chi_{n}\right\rangle$$

and the operator *A* ^(+)^ is given by

3 $$A^{\left(+\right)}=\frac{Ĥ}{N+1}-\frac{\left(ĤP+PĤ\right)}{2}+\frac{\left(PV+VP\right)}{2}-VG_{P}^{\left(+\right)}V$$

In the above equations, $\left|S_{{\mathrm{k⃗}}_{i(f)}}\right\rangle$ is an eigenstate of the unperturbed Hamiltonian *H*_0_ = *H*_*N*_ + *T*_*N*+1_ and is given by the product of a target state and a plane wave with *k⃗*_*i(f)*_ representing the momentum of the free incident (scattered) electron. In the definition of *H*_0_, *H*_*N*_ represents the target Hamiltonian and *T*_*N*+1_ corresponds to the kinetic energy operator of the incident electron. *V* is the interaction potential between the incident electron and the target’s electrons and nuclei; *Ĥ* = *E* – *H*, where *E* is the total collision energy and *H* is the (*N*+1)-electron Hamiltonian in the fixed nuclei approximation; $G_{P}^{\left(+\right)}=PG_{0}^{\left(+\right)}$ is the free-particle Green’s function projected on the *P*-space and *P* is a projection operator onto the open-channel space of the target given by the expression:

4 $$P=\overset{N_{\mathrm{open}}}{\underset{l=1}{\sum }}\left|\Phi_{l}\right\rangle \left\langle \Phi_{l}\right|$$

where |Φ_*l*_⟩ represents the target states, which can be the ground state or any electronically excited state of the target molecule. *N*_open_ refers to the number of the energetically accessible channels which are considered as open in the calculations. As the incident electron energy increases, more channels become energetically accessible.

The |χ_*m*_⟩ represents a basis set of (*N* + 1)-electron Slater determinants (CSFs—configuration state functions), which are constructed as spin-adapted products of target states and single-particle scattering orbitals:

5 $$\left|\chi_{mn}\right\rangle =A\left[\left|\Phi_{m}^{s}\right\rangle ⊗\left|\phi_{n}\right\rangle \right]$$

where A is the antisymmetrization operator, $\left|\Phi_{m}^{s}\right\rangle$ represents the molecular target state, where $\left|\Phi_{1}^{0}\right\rangle$ indicates the ground state obtained at the Hartree–Fock level and $\left|\Phi_{m}^{s}\right\rangle$ (*m* ≥ 2) represents an *N*-electron Slater determinant obtained by performing single excitations from the occupied valence (hole) orbitals of the ground (reference) state to a set of unoccupied (particle) orbitals with spin *s* (*s* = 0 for singlet states or *s* = 1 for triplet states). |ϕ_*n*_⟩ is a scattering orbital.

## 3 Computational Details

The ground state geometry of the pyrimidine molecule was optimized in the C_2*v*_ point group, using second-order Møller-Plesset perturbation theory (MP2) and the aug-cc-pVDZ basis set, as implemented in the GAMESS^33^ computational package. In both bound state and scattering calculations, the pseudopotentials of Bachelet et al.^31^ were used to represent the nuclei and core electrons of the carbon and the nitrogen atoms. The single-particle basis set employed in the calculations consisted of 5*s*5*p*2*d* Cartesian Gaussian (CG) functions, generated according to ref 28, with exponents listed in Table 1. For the hydrogen atom, we used the Dunning^34^ 4s/3s basis set, with the addition of a *p*-type function with exponent of 0.75.

**Table 1:** Exponents of the Uncontracted Cartesian Gaussian Functions Used for Carbon (C) and Nitrogen (N) Atoms in the Present Calculations Performed with the SMC Method

Type	C	N
*s*	12.49628	17.56734
*s*	2.470286	3.423615
*s*	0.614028	0.884301
*s*	0.184028	0.259045
*s*	0.039982	0.055708
*p*	5.228869	7.050692
*p*	1.592058	1.910543
*p*	0.568612	0.579261
*p*	0.210326	0.165395
*p*	0.072250	0.037192
*d*	0.603592	0.403039
*d*	0.156753	0.091192

The ground state of the target molecule was described using the Hartree–Fock method, while the excited states were described according to the minimal orbital basis for the single configuration interaction (MOB-SCI)^29^ strategy. The following steps were then carried out to proceed with the calculations: *i*) improved virtual orbitals^35^ (IVOs) were used to represent the particle and scattering orbitals; next, a full single configuration interaction (FSCI) calculation was performed, yielding 3015 singly excited Slater determinants (or 3015 particle-hole pairs), resulting in 6030 electronically excited states, consisting of 3015 singlet states and 3015 triplet states; *ii*) from the 6030 electronically excited states generated in the FSCI calculation, which serves as our reference, we selected the 200 lowest-energy states for the scattering calculations. To describe these 200 excited states, we used 147 particle-hole pairs, resulting in a total of 294 electronically excited states, consisting of 147 singlet states and 147 triplet states. When selecting the hole-particle pairs for the MOB-SCI scheme, we ensured that the energy values obtained within this strategy would maintain at least 90% of agreement with those obtained from the FSCI calculation.

In Table 2, we present the vertical excitation energies obtained from the FSCI calculation and the MOB-SCI strategy for the first 38 electronically excited states, both singlet and triplet, of the pyrimidine molecule. The energies obtained from the MOB-SCI calculations show agreement ranging from excellent (with a difference of 0.1 eV) to reasonable (with a difference of up to 1.0 eV) when compared to the FSCI spectrum. However, in some cases, there are changes in the order of the states. We compared the spectrum obtained using the MOB-SCI strategy with the theoretical^17,36^ and experimental^37,38^ results available in the literature. It is observed that the lower-energy states are in good agreement with both the theoretical results and experimental data. Figure 2 presents a schematic representation of the 294 electronically excited states obtained in the calculations, along with the strategy adopted for the MOB-SCI calculation. The colored lines indicate the different levels of multichannel coupling used in the scattering calculations.

**Table 2:** Vertical Excitation Energies (in eV) for the First 38 Excited Electronic Singlet and Triplet States Obtained from FSCI and MOB-SCI Calculations^a^

State	FSCI	MOB-SCI	Ref 17	Ref 17	Ref 36	Ref 37	Ref 38
1^3^ *A*_1_	3.58	3.97	4.00	3.97	4.45	4.00	
1^3^ *B*_1_	4.59	4.71	4.54	4.54	3.05	3.8	
1^3^ *B*_2_	4.93	5.42	5.12	5.08	4.50	4.80	
2^3^ *A*_1_	5.36	5.51	5.27	5.23		5.10	
1^1^ *B*_2_	5.78	6.10	5.13	5.09	5.44	5.30	5.22
1^3^ *A*_2_	5.57	6.15	5.24	5.29	3.46	4.40	
1^1^ *B*_1_	6.28	6.51	4.99	4.97	3.44	4.30	4.183
2^3^ *B*_1_	6.37	6.59	7.07	7.05	4.6	5.70	
1^1^ *A*_2_	6.44	6.82	5.63	5.63	3.67	4.80	
2^3^ *B*_2_	7.31	7.36	7.42	7.37			
2^1^ *A*_1_	6.71	7.42	8.34	8.27	6.35	6.80	6.69
2^3^ *A*_2_	6.86	7.42	6.45	6.43	4.20	5.40	
2^1^ *B*_2_	7.50	7.53	8.53	8.46	6.55		
2^1^ *A*_2_	7.35	7.80	6.71	6.67	4.65	5.90	
3^3^ *B*_2_	7.63	8.12	8.07	8.02			
3^3^ *A*_1_	8.04	8.31	7.54	7.50			
3^3^ *B*_1_	7.90	8.41	9.37	9.33			
4^3^ *B*_2_	8.37	8.44	10.31	10.3			
3^3^ *A*_2_	8.43	8.46	9.04	9.03			
3^1^ *B*_2_	8.13	8.47	10.29	10.22	7.42	7.60	7.478
3^1^ *A*_2_	8.49	8.5	9.2	9.18			
4^1^ *B*_2_	8.51	8.55	10.51	10.48	7.50		
3^1^ *A*_1_	8.32	8.65	8.84	8.76	7.40		
4^3^ *A*_1_	8.62	8.69					
4^3^ *B*_1_	8.52	8.74	10.26	10.22	7.42		
4^3^ *A*_2_	8.69	8.81	10.26	10.21			
2^1^ *B*_1_	8.63	8.83	7.23	7.21		6.10	6̃.00
4^1^ *A*_2_	8.85	8.91					
5^3^ *B*_2_	8.99	9.09					
5^3^ *A*_1_	8.94	9.21					
4^1^ *A*_1_	8.81	9.31	10.18	10.10	7.19	7.60	7.478
5^1^ *B*_2_	9.24	9.31					
6^3^ *B*_2_	9.30	9.37					
6^1^ *B*_2_	9.44	9.49					
5^3^ *A*_2_	9.26	9.56					
5^1^ *A*_2_	9.56	9.60					
3^1^ *B*_1_	8.74	9.67	10.46	10.41	7.42		
7^1^ *B*_2_	9.63	9.67					
5^1^ *A*_1_	9.32	9.68					
6^1^ *A*_2_	9.66	9.77					

a We compared our results with theoretical data obtained by Mašín *et al.*,^17^ who employed the state-averaged complete active space self-consistent field (SA-CASSCF) method with two different basis sets—cc-pVDZ and 6–311+G. We also considered the calculations by Stener *et al.*,^36^ performed using time-dependent density functional theory (TD-DFT), as well as the experimental data from Fischer *et al.*,^37^ obtained through UV absorption spectroscopy, and from da Silva *et al.*,^38^ who used vacuum ultraviolet (VUV) photoabsorption and electron energy loss spectroscopy (EELS).

**Figure 2:**
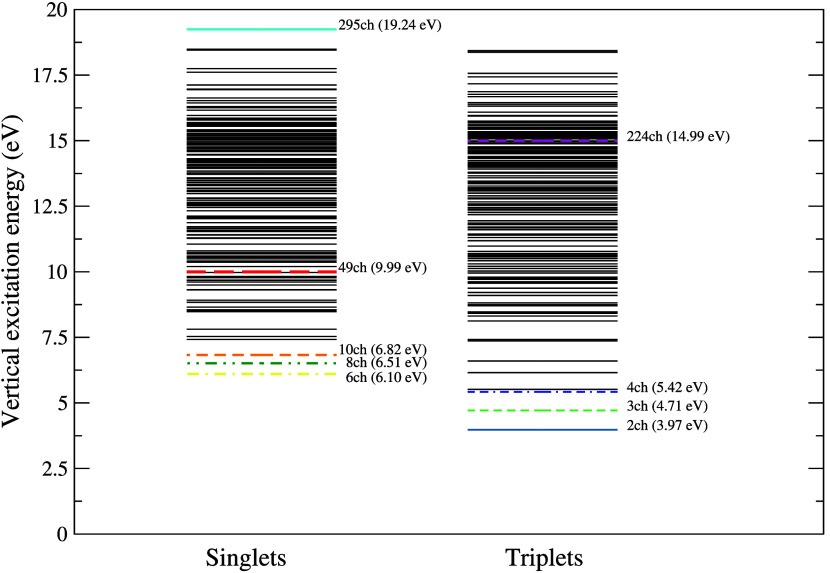
Schematic representation of the vertical excitation energies (in eV) of the 294 electronically excited states of pyrimidine obtained with the MOB-SCI calculation and different levels of channel coupling employed in the present scattering calculations performed by means of the SMC method. Solid steel blue line, 2ch; dashed green line, 3ch; double-dashed-dotted blue line, 4ch; dashed-dotted yellow line, 6ch; double-dotted-dashed dark green line, 8ch; dashed orange line, 10ch; dashed red line, 49ch; dashed magenta line, 224ch; solid cyan line, 295ch.

The same hole-particle pairs used to construct the active space in the MOB-SCI strategy were also employed in forming the CSF space to describe the polarization of the molecular target. The number of CSFs obtained for each symmetry were: 14227 for *A*_1_ symmetry, 13875 for *A*_2_ symmetry, 14215 for *B*_1_ symmetry, and 13767 for *B*_2_ symmetry. The scattering calculations in this work were performed at different levels of channel coupling, meaning that, depending on the electron’s incident energy, different channels are considered open (i.e., different electronically excited states become accessible as the energy increases) in the *P* operator (Equation 4). However, we present here only the cross sections associated with the best coupling level (where the channels are considered open) for each impact energy considered. To distinguish between the different strategies, we adopted the nomenclature used in ref 39, denoted as *N*_open_ch, where *N*_open_ is the number of open channels considered in the *P* operator. As shown in Figure 2, the coupling levels used in this work are 2ch, 3ch, 4ch, 6ch, 8ch, 10ch, 49ch, 224ch, and 295ch.

Pyrimidine is a polar molecule, with a calculated permanent dipole moment of 2.50 D in the present work, which is in good agreement with the experimental value of 2.33 D.^40^ Thus, we used the Born-closure procedure to describe the long-range potential generated by the molecule’s dipole moment. The Born-closure procedure combines the scattering amplitude obtained with the SMC method and the scattering amplitude of the dipole potential, calculated in the first Born approximation (FBA). This approach is applied to improve the description of the DCSs at small scattering angles. In summary, the scattering amplitude obtained with the SMC method is expanded in partial waves up to a specific value of *l*_SMC_, while the dipole potential scattering amplitude is calculated using the FBA and also expanded in partial waves. The two amplitudes are then combined, where the SMC amplitude describes partial waves up to *l*_SMC_, and the dipole amplitude describes partial waves from *l*_SMC_ + 1 to ∞. The *l*_SMC_ value is chosen by comparing the DCSs obtained with and without the Born-closure procedure, which coincide for angles above approximately 20°. Further details on the method can be found in ref 32. The selection of *l*_SMC_ values depends on the incident electron energy. The chosen values were: *l* = 1 for the interval from 0.1 to 0.4 eV, *l* = 2 from 0.5 to 0.7 eV, *l* = 3 from 0.8 to 3.0 eV, *l* = 4 from 3.1 to 3.9 eV, *l* = 5 from 4.0 to 4.6 eV, *l* = 6 from 4.7 to 5.4 eV, *l* = 7 from 5.5 to 7.0 eV, *l* = 8 from 7.1 to 8.5 eV, *l* = 8.6 from 9.5 to 9.9 eV, and *l* = 10 from 10.0 to 50.0 eV.

To determine the total cross section (TCS), we calculated the ionization cross section and added it to the contributions from the elastic and electronically inelastic cross sections obtained using the SMC method. With this in mind, we used the BEB model^30^ to obtain the total ionization cross section (TICS). The widely used BEB model provides a straightforward analytical formula for determining the ionization cross section resulting from electron impact on atoms and molecules. Within this model, the ionization cross section for the *i*-th molecular orbital is defined as

6 $$\sigma_{i}\left(t_{i}\right)=\frac{4\pi a_{0}^{2}N_{i}{\left(R/B_{i}\right)}^{2}}{t_{i}+u_{i}+1}\times \left[\frac{\ln\left(t_{i}\right)}{2}\left(1-\frac{1}{t_{i}^{2}}\right)+1-\frac{1}{t_{i}}-\frac{\ln\left(t_{i}\right)}{t_{i}+1}\right]$$

where *B*_*i*_ is the binding energy of the electron of the *i*th molecular orbital, *t*_*i*_ = *E*/*B*_*i*_, *u*_*i*_ = *U*_*i*_/*B*_*i*_, where *E* is the kinetic energy of the incident electron, *U*_*i*_ is the average kinetic energy of the *i*th molecular orbital, *a*_0_ is the Bohr radius, *R* is the Rydberg energy and *N*_*i*_ is the occupation number of the *i*-th molecular orbital. The TICS is calculated by summing the ionization cross sections of all orbitals involved in the process, i.e.,

7 $$\sigma_{BEB}=\overset{N_{occ}}{\underset{i=1}{\sum }}\sigma_{i}\left(t_{i}\right)$$

where *N*_occ_ is the number of occupied molecular orbitals of the molecular target. The parameters required for the calculations were obtained in the equilibrium geometry in the ground state in a Hartree–Fock level calculation performed with the aug-cc-pVDZ basis set implemented in the GAMESS^33^ computational package. The value obtained for the ionization threshold was 10.27 eV, a good agreement with the experimental results of 9.73 eV.^60^

## 4 Results and Discussion

### 4.1 Elastic Scattering Resonances

Figure 3 shows our elastic ICS for impact energies ranging from 0.1 to 50 eV compared to those available in the literature. We present results for the calculations considering 1 to 295 energetically accessible channels, without and with the Born-closure procedure. We identified three resonant structures, centered at 0.62 $\left(\pi_{1}^{*}\right)$ and 0.82 $\left(\pi_{2}^{*}\right)$ eV, and approximately at 4.62 $\left(\pi_{3}^{*}\right)$ eV. The first resonance is associated with *A*_2_ symmetry, while the latter two are associated with *B*_1_ symmetry. Due to the presence of a structure at 5.10 eV, we performed the diagonalization of the scattering Hamiltonian to determine the eigenvalue associated with the $\pi_{3}^{*}$ resonant state. This approach has been employed by the group to map and characterize the resonances, with further details available in refs 41–43 . The diagonalization revealed a resonant state located at 4.73 eV, along with a Dyson orbital of π* character, indicating that the structure observed at 4.62 eV in our calculations corresponds to a physical resonance and suggesting that the structure observed at 5.10 eV is possibly a threshold effect resulting from the opening of nearby channels. In fact, this structure is located between the thresholds of the 1^3^ *B*_1_ (4.71 eV) and 1^3^ *B*_2_ (5.42 eV) states, suggesting that it may originate from the opening of these channels. This type of structure can be understood as a threshold effect, in which the opening of a new inelastic channel induces a sudden change in the cross section, and it has been observed in other studies involving collisions not only with electrons,^44,45^ but also with other particles, as reported in refs 46–49 . This phenomenon, known in the literature as the Wigner-cusp effect,^50^ arises from the unitarity and analyticity of the scattering matrix near the opening of a reaction channel. As a result, the cross section may exhibit a cusp-like anomaly or a smooth discontinuity at energies in the vicinity of the threshold.

**Figure 3:**
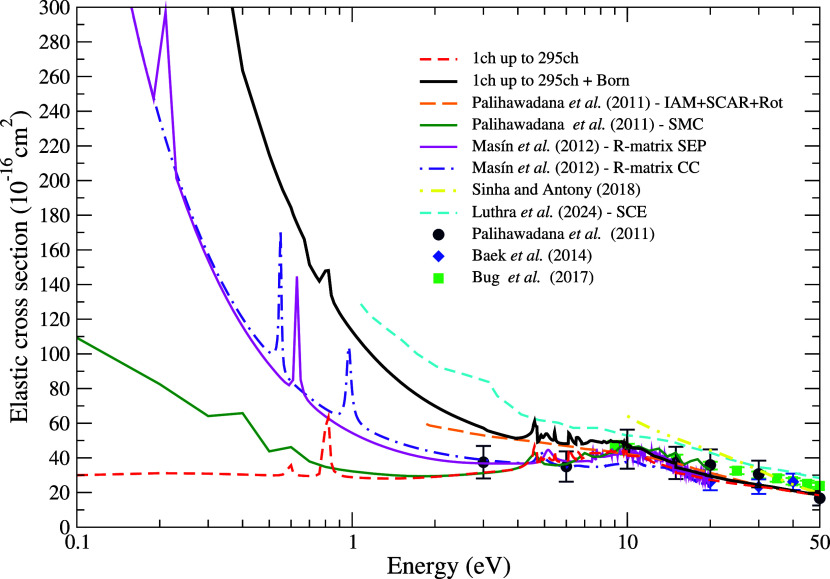
Integral cross section for elastic electron scattering by pyrimidine. See the text for further discussion.

As already observed in previous works performed by our group,^41,42,56^ these structures may appear in cross sections due to the way of how the coupling between the electronic channels are taken into account in the SMC method, which is through the projection operator *P*. In other methods, the distinct way of treating the channel coupling may attenuate or even smooth out such effects. To conclude, it is worth noting that Č*i*žék et al.^55^ reported the difficulties faced by experimentalists in measuring these structures, since unwanted electric or magnetic fields (even very weak ones) in the collision region can prevent very low energy electrons from reaching the detector. Experimentalists also face difficulties in resolve individual channels in their data, which can smooth out the Wigner cusps. As observed in our calculations, the cross section becomes more sensitive when the channels are opened one by one. When states with similar energies are grouped together, which is the strategy adopted at higher energies (high density of states), a smoother cross section is obtained. Machacek et al.^49^ conducted studies on positron collisions with isoelectronic atoms and molecules, namely Ne, H_2_O, NH_3_, and CH_4_, investigating the presence of a Wigner cusp at the threshold of opening the elastic channel relative to the positronium formation channel. The authors identified a cusp in the scattering cross section of the Ne atom, but were unable to observe cusps in the other studied molecules. They attributed the absence of these cusps to the presence of vibrational modes, which suppress the manifestation of the cusp in the region where one channel opens to another. Khakoo et al.^44^ also investigated electron-impact excitation of the a″ Σg+1 state of the N_2_ molecule. The authors suggest that the appearance of cusp-like structures may be associated with coupling between excitation channels with the same symmetry, that is, when the ground and excited states share the same symmetry. This behavior was observed in studies on the atomic targets He,^51^ Hg,^52,53^ and Ba.^44^ Following the suggestion of the authors,^44^ we investigated the behavior near the thresholds. In Figure 4 we present our ICS in the energy range from 0 to 7 eV, highlighting the Wigner-cusps type structures associated with the opening of the 2ch, 3ch, 4ch, 6ch, and 8ch channels. These characteristic features appear precisely at the opening of the respective channels, in agreement with the structures investigated by Hotop et al.^54^

**Figure 4:**
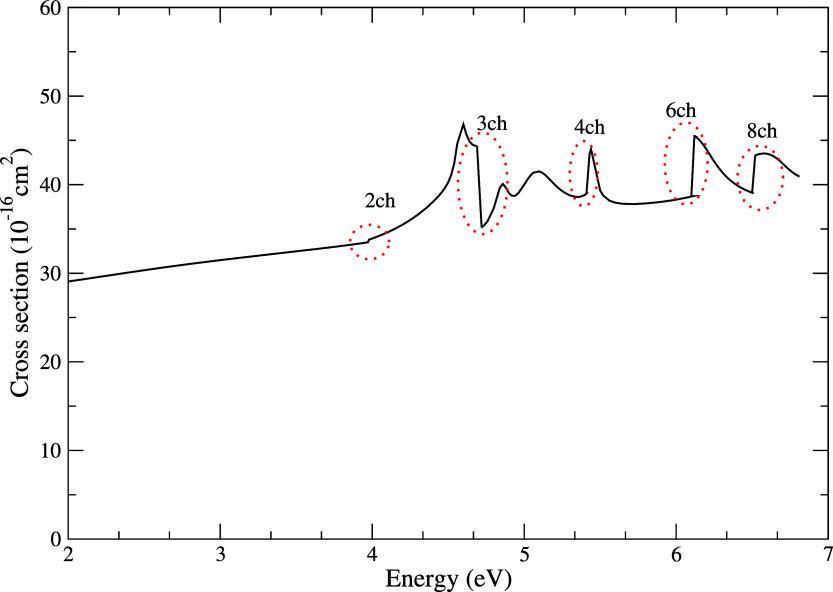
Elastic integral cross section for electron scattering by the pyrimidine molecule showing the Wigner-cusps. The red dashed circles highlight the region in which the opening of the channels: 2ch, 3ch, 4ch, 6ch and 8ch occurs. See the text for further discussion.

The $\pi_{1}^{*}$ and $\pi_{2}^{*}$ are shape resonances while $\pi_{3}^{*}$ is a mixture of shape and core-excited resonances, in agreement with the assignments of Palihawadana et al.^16^ and Mašín et al.^17^ Current results and those available in the literature on the position of the resonant structures are summarized in Table 3. The resonance positions obtained by Palihawadana et al.,^16^ using the SMC method, show an inversion between the first and second resonances compared to ours. A difference of 0.01 eV is observed for the first resonance, 0.44 eV for the second, and 0.02 eV for the third, indicating a fair agreement between both calculations.

**Table 3:** Comparison between the Positions of the Resonances (in eV) Observed in the Elastic Scattering of Electrons by the Pyrimidine Molecule

	$\pi_{1}^{*}$	$\pi_{2}^{*}$	$\pi_{3}^{*}$
Theoretical results			
Present work	0.62	0.82	4.62
Palihawadana et al.^16^	0.63	0.38	4.60
Mašín et al.^17^ (SEP)	0.21	0.63	5.15
Regeta et al.^13,14^ (CC)	0.53	0.96	4.78
Experimental results			
Regeta et al.^13,14^	0.27	0.70	4.35
Nenner and Schulz^11^	0.25	0.77	4.24
Modelli et al.^12^	0.39	0.82	4.26

Regarding to the results obtained by Mašín et al.,^17^ using the R-matrix method, the two lowest-energy resonances identified by these authors are located at lower energies in comparison to our results, with differences of 0.41 and 0.44 eV for the first and second resonances, respectively. The third resonance, however, appears at a higher energy compared to our results, with a difference of 0.53 eV. Concerning the resonance positions obtained by Regeta et al.,^13,14^ who also used the R-matrix method, the first resonance is located at a lower energy compared to our calculations, with a difference of 0.09 eV. The second and third resonances, however, are positioned at higher energies compared to ours, with differences of 0.14 and 0.16 eV, respectively. Our results are in fair agreement with the resonance positions experimentally obtained by Regeta et al.,^13,14^ Nenner and Schulz,^11^ and by Modelli et al.^12^ The difference between the positions reported by these experiments and ours ranges from 0.23 to 0.37 eV for the first resonance, from 0.05 to 0.12 eV for the second resonance, and from 0.27 to 0.38 eV for the third resonance.

### 4.2 Elastic Scattering Cross Sections

Regarding the magnitude of the ICS show in Figure 3, we obtained excellent agreement with Palihawadana’s et al.^16^ results, also obtained with the SMC method, but using a different polarization scheme. Above 10 eV, our ICS shows a significant reduction in magnitude, which is due to the increase in the number of energetically accessible channels considered open in our calculations, leading to competition for the cross section flux. This competition arises because, as the number of available channels increases (such as electronic excitations included in our calculations), the probability flux that was previously concentrated solely in the elastic channel becomes distributed among multiple processes. As a result, the cross section associated with each individual channel—such as the elastic one—tends to decrease. In the calculations by Palihawadana et al.,^16^ on the other hand, only the elastic channel is considered open, which prevents this redistribution of the flux. Our results agree well with the IAM+SCAR+Rot results above 10 eV, where the independent-atom model usually describes the electron-molecule interaction more accurately. Mašín et al.^17^ presented results at two levels of calculations, SEP and close-coupling (CC), both without Born-correction. The SEP calculations shows several structures above 8 eV which, as reported by the Mašín et al.,^17^ are nonphysical and result from channels energetically accessible but kept closed at this level of calculation. In contrast, the CC calculations present a smoother cross section, as these channels are treated as open. Our result without applying the Born-closure procedure is in better agreement with the SEP calculation from Mašín et al.^17^ up to 10 eV. For the energies below 9.98 eV, the magnitude of the cross section obtained by Mašín et al.^17^ at the CC level is lower than our results, as only 10 channels (or less) are open in our calculations, whereas Mašín et al.^17^ considered 21 open channels. Above this energy, the differences between the calculations become small. Concerning the results obtained by Sinha and Antony,^24^ we observe a significant difference in the magnitude of the integral cross section compared to our results. The SCOP method used by the authors calculates the cross section based on a complex potential, consisting of a real part, which describes the elastic scattering, and an imaginary part, which accounts for the loss of flux due to all possible inelastic processes. We believe that the elastic part was not accurately described, as evidenced by the discrepancy between the authors’ results and those available in the literature.

Regarding the experimental data reported by Palihawadana et al.,^16^ good agreement is observed for energies above 10 eV, within the experimental uncertainties. For energies below 10 eV, the ICS reported by the authors shows good agreement with our results without applying the Born correction. The experimental ICS is obtained by integrating the DCS; however, prior to integration, the data must be extrapolated to both very small and very large angles—and it is this extrapolation that introduces the differences observed in the ICS. As discussed by Mašín et al.,^17^ when the experimental DCS are integrated only over the angular range where measurements are available and compared with the theoretical results (including the Born correction) integrated over the same range, the agreement between theory and experiment is excellent. Finally, with respect to the results of Luthra et al.,^25^ we observe a difference in the magnitude of the cross sections compared to those obtained in our work, possibly due to differences in how polarization effects are treated in the two methods. The calculations presented by the authors include corrections associated with long-range dipole effects, and the cross sections are obtained through the use of model potentials. For the data from Baek et al.,^20^ our results are in excellent agreement, within the error margins specified by these authors. However, compared to the data obtained by Bug et al.,^21^ our results are below the reported values, which may be attributed to the extrapolation used to determine the cross section. The authors highlight the sensitivity of the extrapolated cross section derived from the DCSs, which may explain the observed discrepancies.

In Figure 5, we present the elastic DCSs for electron scattering by the pyrimidine molecule at the energies of 6, 10, 15, 20, 30, and 50 eV. The DCSs correspond to the best coupling levels for each energy: 4 channels at 6 eV, 49 channels at 10 eV, 224 channels at 15 eV, and 295 channels at 20, 30, and 50 eV. Additionally, the Born-closure procedure was applied to account for the long-range dipole moment and, as expected, gives rise to a significant increase in the DCS magnitude at low scattering angles. We compared our results with those available in the literature. Regarding the theoretical results of Palihawadana et al.,^16^ who also used the SMC method, we observed good agreement between 15° and 30° and above 120° at 6 eV. Below 15°, our calculations reflect the correction for the long-range potential caused by the dipole moment. Although the effect of multichannel coupling is not significant in this energy range, the role of polarization effects becomes evident, as reflected in the good agreement with the experimental data also presented by Palihawadana et al.^16^ The discrepancy between 30° and 120° is attributed to differences in the polarization criteria used in both calculations. At energies of 10, 15, 20, and 30 eV, the effects of multichannel coupling are clearly evident, with a significant reduction in the cross section magnitude compared to the theoretical results obtained by Palihawadana et al.,^16^ considering only one open (elastic) channel.

**Figure 5:**
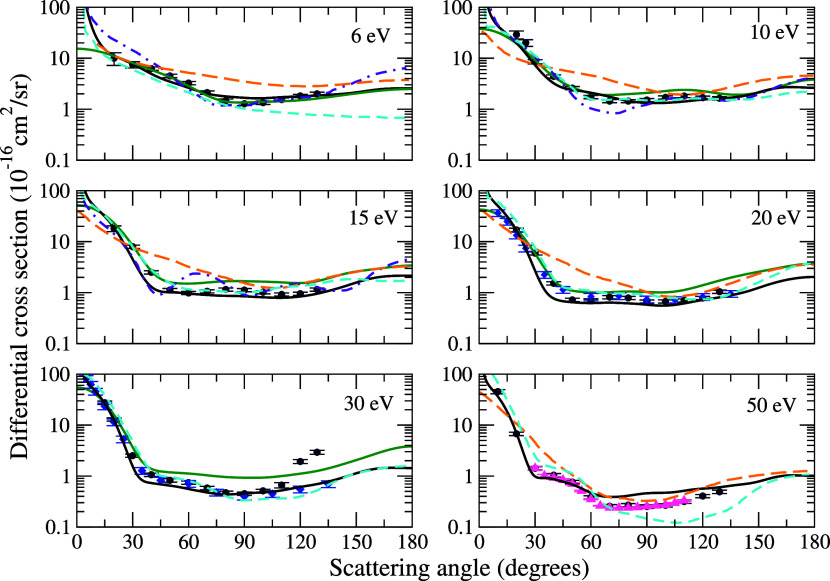
Differential cross sections for elastic electron scattering by pyrimidine at the impact energies of 6, 10, 15, 20, 30, and 50 eV. Solid black line, present SMC with Born-closure results obtained according to the best multichannel coupling scheme (4ch at 6 eV, 49ch at 10 eV, 224ch at 15 eV, 295ch at 20 eV, 30 and 50 eV); solid dark green line, SMC results from Palihawadana et al.;^16^ dashed orange line, IAM+SCAR+Rot results from Palihawadana et al.;^16^ dotted-dashed violet line, R-matrix results from Mašín et al.;^17^ dashed cyan line, SCE results from Luthra et al.;^25^ navy blue circles, experimental measurements reported by Palihawadana et al.;^16^ blue diamonds, experimental measurements reported by Baek et al.;^20^ pink triangle, experimental measurements reported by Maljković et al.^15^ See the text for further discussion.

The results of Palihawadana et al.,^16^ employing the IAM+SCAR+Rot method and considering rotational excitations, do not match our DCSs in the energy range considered in the present study. The authors reported that the method works well at high and intermediate energies, but fails at low energies. Comparison with the results of Mašín et al.^17^ shows good agreement at 6 eV, where the contribution due to the elastic channel dominates. However, at higher energies (10 and 15 eV), the importance of inelastic channels becomes evident, and the neglect of these channels in Mašín’s et al.^17^ calculations gives rise to a pronounced oscillatory pattern that compromises the agreement with our results. In relation with the experimental data of Palihawadana et al.^16^ at 6 eV there is a good agreement for angles below 30° and above 120°. However, for intermediate angles, where polarization is crucial, previous SMC result from ref 16 demonstrates a better fit. Above 10 eV, the influence of multichannel coupling is dominant, and our results show better agreement with the experimental data. At 30 eV, we observe excellent agreement with previous data up to 120°. Above this angle, however, the results of Palihawadana et al.^16^ show a sharp increase. Baek et al.^20^ compared the data in this energy range and highlighted that, at 120°, Palihawadana’s et al.^16^ values are 3.5 times higher than his, which was unexpected, as a similar angular dependence is not observed at either lower or higher electron energies. This behavior is also reflected in our results. Finally, at 50 eV, the agreement is excellent for angles below 60°, with a small discrepancy in terms of magnitude for larger angles, which can be attributed to the influence of other channels not considered in our calculations, which may contribute to the reduction of the cross section. The same comments also apply in the comparison with the data obtained by Maljković et al.^15^ Our results at 20 and 30 eV show excellent agreement with the experimental data of Baek et al.,^20^ especially for angles greater than 120°, the angular range in which our results diverge from those obtained by Palihawadana et al.^16^ With respect to the results obtained by Luthra et al.,^25^ at an energy of 6 eV, the authors’ calculations show good agreement in the behavior of the cross section for scattering angles below 60°. For larger angles, however, we observe a discrepancy relative to our results, possibly associated with polarization effects, which are not adequately described by the use of model potentials, as discussed by the authors themselves. At 10 eV, good agreement between the results is observed. At 15 and 20 eV, there is a small difference in the magnitude of the cross sections, with the values reported by the authors being slightly higher than ours. At 30 eV, the results are again in good agreement, whereas at 50 eV the results of Luthra et al.^25^ begin to diverge from ours.

Figure 6 presents the elastic excitation function (EEF) for electron scattering by the pyrimidine molecule at scattering angles of 90°, 120° and 135°. Our results are compared with two theoretical data sets reported by Regeta et al.:^14^ the first obtained using the SMC method, originally calculated by Palihawadana et al.,^16^ and the second by Mašín et al.^17^ using the R-matrix method. Additionally, we compare our results with the experimental data from Palihawadana et al.,^16^ considering both the EEFs and the DCSs, as well as with the experimental data reported by Regeta et al.^14^ At 90°, the three resonant structures previously discussed in the ICS are clearly observed. A discrepancy between the theoretical results from ref 14 is noted in the 3–7 eV range, which may be attributed to the presence of the third resonance and threshold effects accounted for in our calculations. Above 7 eV, our results lie below the other theoretical predictions, likely due to the larger number of channels considered in our model, which results in better agreement with the experimental data. With respect to the experimental data,^16^ our results show overall good agreement, except between 4 and 7 eV, where they are higher than the measured values. This discrepancy may be related to the presence of several structures in this energy region. The first two pronounced features correspond to shape resonances, while the third, located at 4.62 eV, is attributed to the $\pi_{3}^{*}$ resonance also identified in our ICS, as discussed before. Additional structures may be associated with core-excited resonances, as predicted by Modelli et al.^12^ and reported in the calculations of Regeta et al.,^14^ with the threshold effects previously mentioned or may be nonphysical structures associated with linear dependency in the set of basis functions. Such structures were identified in our work at approximately 6.30, 6.53, 5.44, 6.15, 6.64, 7.40, and 8.30 eV, associated with the symmetries *ã* ^2^ *A*_1_, *b̃* ^2^ *A*_1_, *c̃* ^2^ *A*_2_, *d̃* ^2^ *B*_1_, *ẽ* ^2^ *B*_1_, *f̃* ^2^ *A*_2_, and *g̃* ^2^ *B*_1_, following the same nomenclature adopted by Regeta et al.^14^ In the experimental data of Regeta et al.,^14^ two structures are observed at 5.55 eV, arising from the symmetries *c̃* ^2^ *A*_2_ and *d̃* ^2^ *B*_1_, while in our results they are found at 5.44 and 6.15 eV; two structures at 6.52 eV, associated with the symmetries *ẽ* ^2^ *B*_1_ and *f̃* ^2^ *A*_2_, while in our results they are located around 6.64 and 7.40 eV; and one structure at 7.45 eV, attributed to the symmetry *g̃* ^2^ *B*_1_, which in our calculations is found around 8.30 eV. The authors’ calculations, however, indicate two additional structures not identified experimentally, located at 5.96 and 6.15 eV, corresponding to the symmetry *A*_1_ (*ã* ^2^ *A*_1_ and *b̃* ^2^ *A*_1_), whereas in our calculations they appear at 6.30 and 6.53 eV. The remaining structures appear at equivalent positions: 6.11, 6.37, 7.11, 7.33, and 8.47 eV. In turn, Modelli et al.^12^ identified two structures at 5.50 eV, related to the symmetries *c̃* ^2^ *A*_2_ and *d̃* ^2^ *B*_1_. At 120°, our results are compared with the experimental data obtained by Palihawadana et al.^16^ Despite the presence of structures in the 4–7 eV range, possibly associated with resonances and threshold effects, excellent agreement with the experiments is observed. At both analyzed angles, the resonant structures show good correspondence with those reported in the literature, especially above 6 eV, where our results coincide with the experimental data. Finally, at 135°, our results exhibit agreement in terms of behavior with the theoretical results of Palihawadana et al.^16^ up to 10 eV. Above this energy, our values lie below those obtained by these authors, which can be attributed to the larger number of open channels considered in our calculations. We also observe good agreement with the theoretical results of Regeta et al.,^14^ with only a small difference in magnitude above 10 eV, also related to the number of open channels considered in our calculations. Regarding the experimental results obtained by Regeta et al.,^14^ our results show good agreement; however, above 10 eV, they align with the experimental data when the authors’ results are shifted by a multiplicative factor of ×1.5 to the left, as shown in Figure 6 (solid red curve).

**Figure 6:**
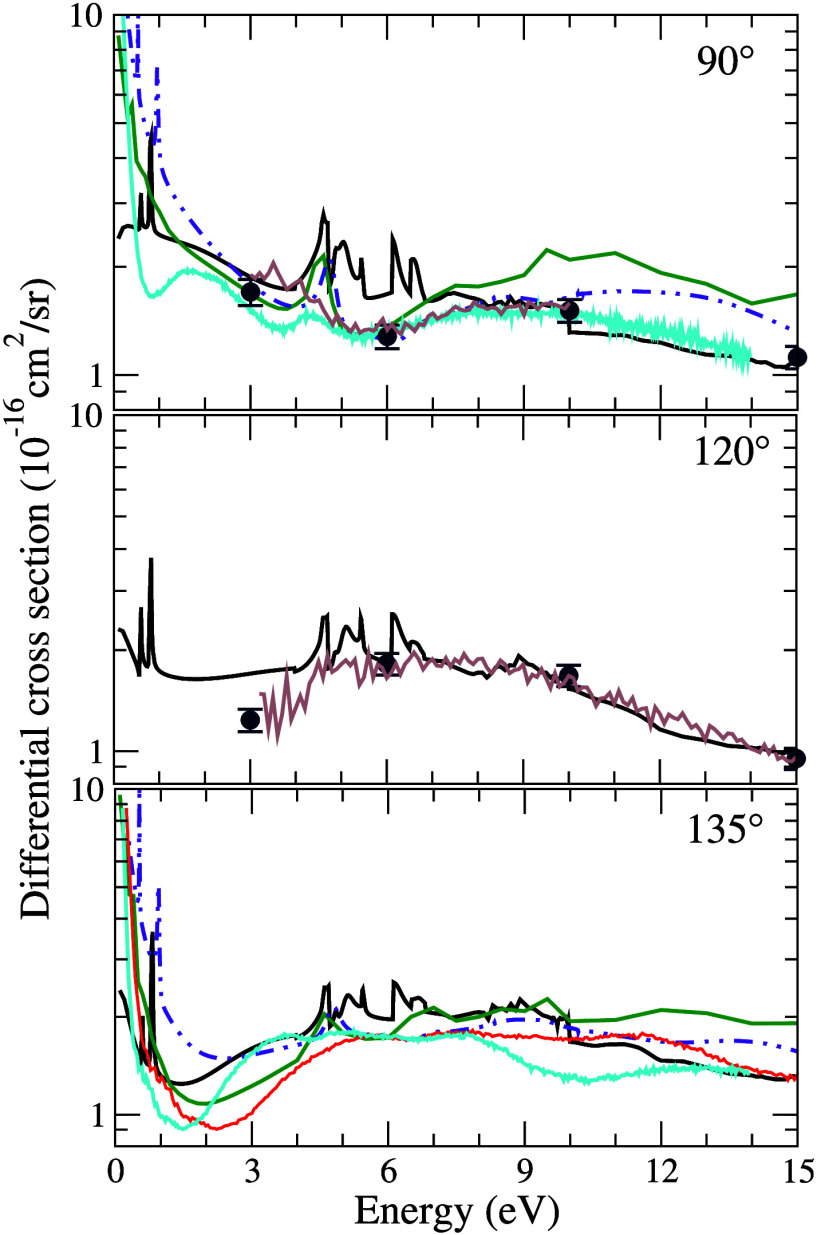
Excitation functions for elastic electron scattering from pyrimidine at the angles of 90°, 120° and 135°. Solid black line, present results; solid green line, theoretical results obtained with the SMC method by Regeta et al.;^14^ double-dotted-dashed indigo line, theoretical results obtained with the R-matrix method by Regeta et al.;^14^ navy blue circles and solid brown line experimental measurements reported by Palihawadana et al.;^16^ solid cyan line, experimental measurements reported by Regeta et al.^14^ and solid red curve experimental measurements reported by Regeta et al.^14^ shifted by a multiplicative factor of 1.5. See the text for further discussion.

### 4.3 Ionization Cross Sections

The TICS for electron impact with pyrimidine calculated using the BEB model from the first ionization threshold (10.27 eV) to 1000 eV is shown in Figure 7. As expected, the curve displays a sharp rise, peaking at around 80 eV, followed by a decrease as the energy increases. Present results are then compared with the theoretical and experimental data available in the literature. Good agreement is observed with the data of Bug et al.,^21^ with our results falling within the error margins reported by these authors. Regarding the theoretical results obtained using a semiempirical model to calculate the TICS and the experimental results, both reported by Wolff et al.,^57^ the agreement is reasonable. Compared to the experimental measurements of Linert et al.^59^ our TICS result displays an acceptable level of accord up to approximately 30 eV, but is in complete disagreement for energies above this value. However, it is worth noting that these data exhibit a flat maximum, a behavior which, as reported by Wolff et al.,^57^ is not characteristic of electron-impact ionization in atoms and small molecules. Finally, we observed a qualitatively similar behavior to the theoretical results reported by Gupta et al.;^58^ however, there is a significant difference in the magnitude of the cross section. Considering that the BEB model usually provides cross sections with agreement within 10% or better with experimental data,^30^ we are confident that we provided an accurate estimate of the total cross section by combining the BEB TICS with the contributions from the elastic and electronically inelastic cross sections obtained by means of the SMC method.

**Figure 7:**
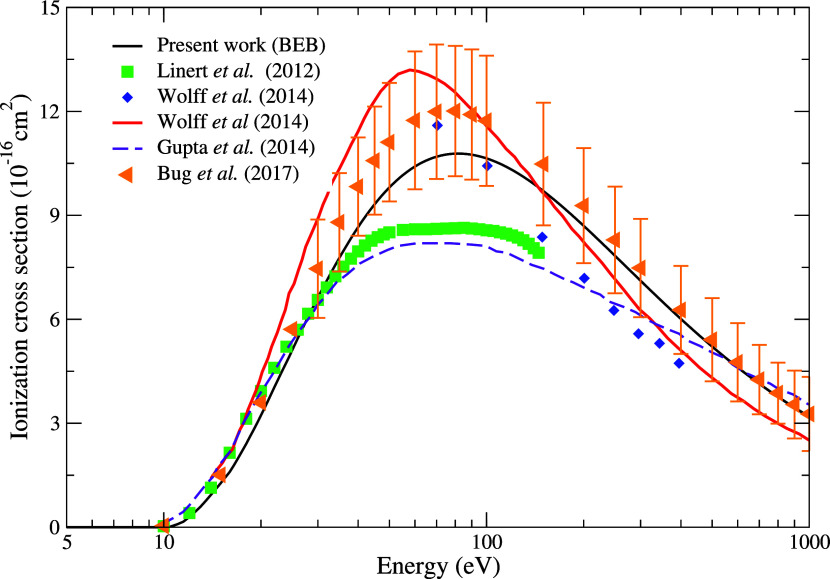
Total ionization cross sections by electron impact of pyrimidine computed with the BEB model compared with literature results. Black solid line, present results; green squares, experimental measurements reported by Linert et al.;^59^ blue diamonds, measurements reported by Wolff et al.;^57^ red solid line, theoretical calculations obtained by Wolff et al.;^57^ dashed violet line, theoretical calculations obtained by Gupta et al.;^58^ orange triangle, experimental measurements reported by Bug et al.^21^ See the text for further discussion.

### 4.4 Inelastic and Total Scattering Cross Sections

Figure 8 shows the excitation cross sections for states that contribute at specific energy loss values. This comparison was performed because of the absence, in the current literature, of results for excitation from the ground state to specific single excited states. The available data, both theoretical and experimental, are presented by energy bands, as the experiments are not able to determine excitation cross sections for state-to-state transitions. The electronic spectrum and the correspondences established by Mašín et al.^17^ regarding the assignments of the experimental data show good agreement, as reported by the authors. Our results are compared with the R-matrix results obtained by Mašín et al.,^17^ and with the experimental data obtained by Levesque et al.^8^ and Regeta et al.^13^ We noticed a difference in magnitude between our results and those obtained by Mašín et al.^17^ The authors also used the energy loss spectrum as a reference, highlighting the states with the most significant contributions to each of the bands. In Figure 8a, it can be seen that the cross sections exhibit similar behavior; however, due to their low magnitude, they are highly sensitive to threshold effects, which explains the various structures observed in the cross section obtained in our calculations that also aligns with the data obtained by Levesque et al.^8^

**Figure 8:**
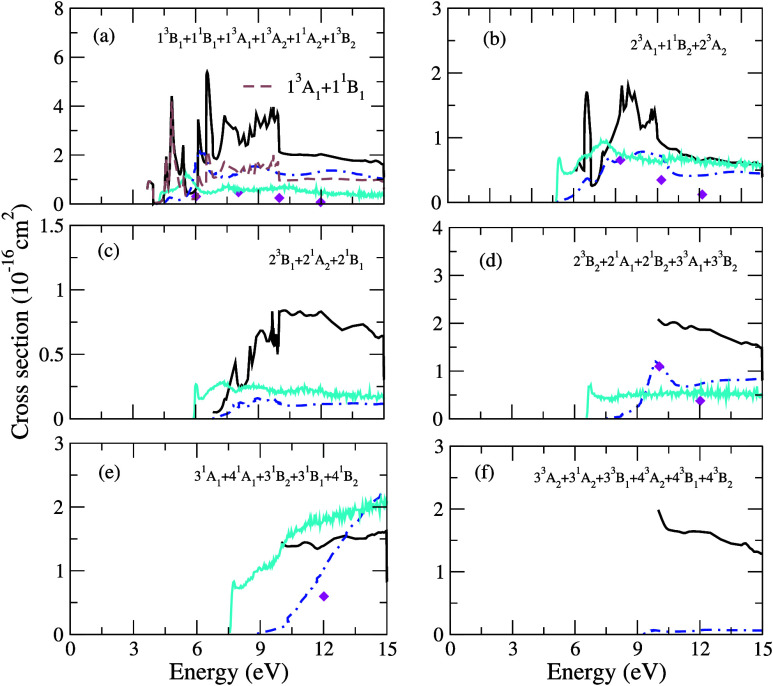
Present excitation cross section summed over groups of states compared to the experimental bands of Levesque et al.^8^ Black solid black and dashed brown lines, present results; dotted-dashed blue line, theoretical calculations of Mašín et al.;^17^ magenta lozenges, experimental measurements reported by Levesque et al.;^8^ solid cyan line, experimental measurements reported by Regeta et al.^13^ a) sum of 1^3^ *B*_1_ + 1^1^ *B*_1_ + 1^3^ *A*_1_ + 1^3^ *A*_2_ + 1^3^ *B*_2_ states; b) sum of 2^3^ *A*_1_ + 1^1^ *B*_2_ + 2^3^ *A*_2_ states; c) sum of 2^3^ *B*_1_ + 2^1^ *A*_2_ + 2^1^ *B*_1_ states; d) sum of 2^3^ *B*_2_ + 2^1^ *A*_1_ + 2^1^ *B*_2_ + 3^3^ *A*_1_ + 3^3^ *B*_2_ states; e) sum of 3^1^ *A*_1_ + 4^1^ *A*_1_ + 3^1^ *B*_2_ + 3^1^ *B*_1_ + 4^1^ *B*_2_ states and f) sum of 3^3^ *A*_2_ + 3^1^ *A*_2_ + 3^3^ *B*_1_ + 4^3^ *A*_2_ + 4^3^ *B*_1_ + 4^3^ *B*_2_ states. See the text for further discussion.

According to our results, the 1^3^ *A*_1_ state contributes significantly to the cross section up to approximately 8.2 eV, beyond which the contribution from the 1^1^ *B*_1_ state becomes predominant. This behavior is also observed in the results of Mašín et al.^17^ (cross section shown in ref 13), where the 1^3^ *A*_1_ state dominates, followed by the 1^1^ *B*_1_ state, which is in full agreement with our findings. Similarly, Regeta et al.^13^ identify these same states as the main contributors to this band. When considering only the contributions from 1^3^ *A*_1_ and 1^1^ *B*_1_ states—represented by the brown dashed line—we observe excellent agreement with the results of Mašín et al.,^17^ where the authors considered five states in their calculations. Nevertheless, a difference in magnitude remains when comparing with the experimental data of Regeta et al.^13^ It is worth noting that when Mašín et al.^17^ considered only two states, a discrepancy in magnitude also appears relative to our results. Similar differences, when comparing with other theoretical calculations,^61,62^ have already been observed in previous works, indicating that further studies are needed to better understand the origin of these discrepancies.

The structure around 4.82 eV can be associated with the first peak observed in both theoretical and experimental results. The features in the 6.14 to 6.60 eV region likely correspond to a single resonance, consistent with that reported in the calculations of Mašín et al.^17^ and Regeta et al.^13^ Likewise, the high-energy structure, above 8 eV, can also be related to the one described by these authors. In Figure 8b, our results also show similarity compared to those obtained by Mašín et al.,^17^ where our findings indicate an equal contribution of the 2^3^ *A*_1_ and 1^1^ *B*_2_ states, which is in agreement with that obtained by the authors. We assign the structure at 6.62 eV to the first peak observed in both theoretical^17^ and experimental^13^ results, associated with a resonance. Above 8 eV, a significant number of structures is observed, and, as highlighted by Regeta et al.,^13^ there is a high density of resonances, which makes the characterization of these structures particularly challenging. In Figure 8c, from 10 eV onward, an abrupt drop is observed in the cross section obtained by Mašín et al.,^17^ although the overall behavior remains similar to our results. For this range, our data indicate that the 2^3^ *B*_1_ state provides the most significant contribution. For the range of energies shown in Figure 8d, the primary contribution comes from the 2^1^ *A*_1_ state, followed by the 2^1^ *B*_2_ and 3^3^ *A*_1_ states, which aligns with the results presented by Mašín et al.^17^ In Figure 8e, our results indicate a predominant contribution from the 3^1^ *A*_1_ and 4^1^ *A*_1_ states, followed by the 3^1^ *B*_2_ state and subsequently the 3^1^ *B*_1_, consistent with the theoretical and experimental results presented by Mašín et al.^17^ Finally, in the cross section presented in Figure 8f, the main contribution comes from the state 4^3^ *A*_2_, followed by the state 4^3^ *B*_1_. The 4^3^ *A*_2_ state is identified as opening at 8.81 eV according to the MOB-SCI strategy. For Mašín et al.,^17^ their calculations indicate that this state opens at 10.21 eV, while the experimental data suggest an energy range between 8.3 and 9.2 eV. The 4^3^ *B*_1_ state, with the highest contribution, is indicated by our calculations as opening at 8.74 eV, whereas Mašín et al.^17^ place it at 10.22 eV. Furthermore, Mašín et al.^17^ highlight the significant influence of Rydberg states in this energy range, emphasizing that their theoretical calculations do not adequately describe these states at higher energies. By following both the criteria for selecting the states that contribute to the energy bands established by Mašin et al.^17^ and those defined by Regeta et al.,^13^ we obtained different cross sections. This indicates that there are aspects of the system’s dynamics that are not yet fully understood or captured by the current models.

Figure 9 shows the estimated TCS (left panel) and the inelastic cross section (right panel) for electron scattering by the pyrimidine molecule. The TCS estimate includes contributions from both elastic and electronically inelastic channels (considering 294 electronically excited states) calculated using the SMC method, combined with the total ionization cross section obtained from the BEB model. It is worth noting that the ionization channel from the BEB model was incorporated *ad hoc* just to estimate the TCS, without affecting the probability flux. Zecca et al.^22^ presented two theoretical results obtained using the IAM+SCAR method, with and without the inclusion of rotational excitations. We observe good agreement between our results and those of Zecca et al.^22^ for energies above 20 eV when rotational excitations are included in their calculations. However, the authors themselves point out that, for energies below 20 eV, their results should not be considered accurate, since the method employed provides a more reliable description for collisions at higher energy regimes. Without accounting for these excitations, Zecca et al.’s^22^ calculations align with the experimental data of Fuss et al.,^23^ which are significantly lower than our results. Bug et al.^21^ attributed this lower magnitude in the data reported by Fuss et al.^23^ to angular resolution limitations, as the polar nature of the pyrimidine molecule tend to produce a significant contribution from rotational excitations in the TCS and strong forward scattering in elastic collisions. The results obtained by Sinha and Antony,^24^ using the SCOP formalism, also show good agreement with ours. For the TCS estimated by Luthra et al.,^25^ as discussed for the other scattering cross sections, we observe a difference in magnitude compared to our results. This discrepancy is again related to the way the model potentials account for polarization effects. The authors emphasize that good agreement is not expected at low energies, due to the strong influence of exchange and polarization effects on the scattering; however, from 45 eV onward, our cross sections show reasonable agreement with the results of Luthra et al.^25^ Regarding the data obtained by Baek et al.^19^ and Bug et al.,^21^ we highlight the excellent agreement with our results, which fall within the experimental error margins reported by these authors.

**Figure 9:**
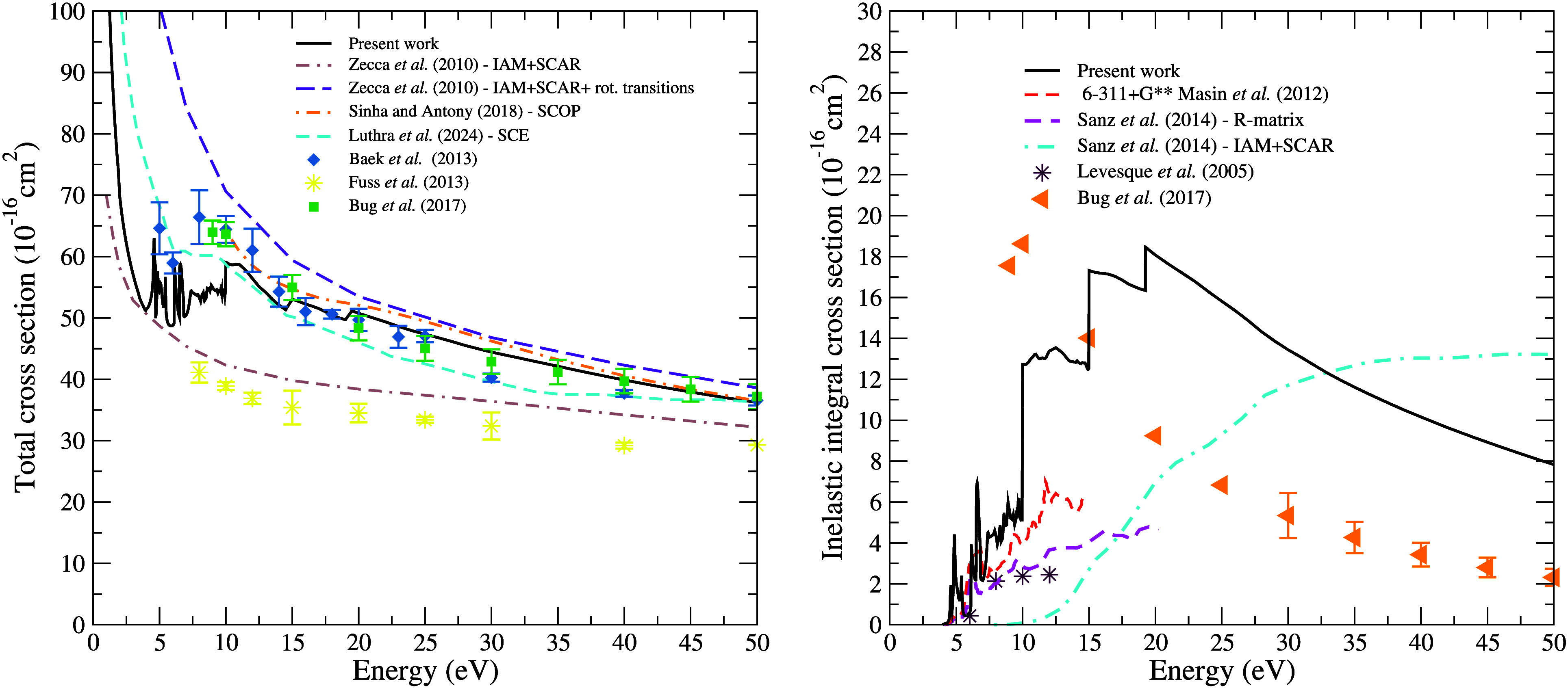
Estimated total cross section (left panel) and electronic excitation cross section (right panel) for pyrimidine. See text for discussion.

The inelastic cross section, shown in the right panel of Figure 9, includes contributions from 294 electronically excited states. The abrupt increase in the cross section at 10, 15, and 20 eV is more pronounced in excitation cross sections than in the elastic one due to the low magnitude involved and can be understood as follows. Below 10 eV, only 10 channels are open and included in the calculations; at 10 eV this number rises to 49, at 15 eV to 224, and at 20 eV reaches 295 channels considered. Due to the high density of excited states, it makes no sense to open these large numbers of channels one by one. As a result, the magnitude of the cross sections changes abruptly. For this reason, although it is associated with the opening of energetically accessible channels, this behavior is nonphysical. We compared our results with those obtained by Mašín et al.^17^ (results presented in the article by Sanz et al.^18^), who used the R-matrix method within the CC approximation, employing two different basis sets and considering 28 electronically excited states in their calculations. It is observed that our results show similar behavior and comparable magnitudes for energies below 10 eV, where few states are accessible. Starting from 10 eV, all 28 electronically excited states considered by Mašín et al.^17^ become accessible; in our calculations, for which 49 states are accessible at this energy. Above 15 eV, more than 200 states become accessible, reaching a total of 294 states. Mašín et al.^17^ discussed the limitations of their calculations in accurately describing states at higher energies, which impacts the magnitude of the cross section. At lower energies, we observe good agreement between the theoretical results and the experimental data from Levesque et al.^8^ The experimental data from Bug et al.^21^ are lower in magnitude than our results, which, in turn, are higher than those obtained by Mašín et al.^17^ Bug et al.^21^ explained that the difference between their data and the theoretical results of Mašín et al.^17^ arises because, in their experiments, rotational excitations cannot be separated from electronic excitations. The results obtained by Sanz et al.,^18^ using the IAM+SCAR method, differ from ours. This discrepancy is likely due to the way in which electronic excitations and ionization processes are included in their calculations, through an absorption potential. We also observe a significant difference between the experimental data from Levesque et al.^8^ and Bug et al.,^21^ indicating that rotational excitations are critical at lower energies due to the dipole moment of the pyrimidine molecule, with their contribution becoming less important at higher energies.

## 5 Conclusions

We presented elastic and electronically inelastic cross sections obtained by means of the SMC method for the scattering of low-energy electrons by the pyrimidine molecule. We also calculated the total ionization cross section by electron impact using the BEB model, obtaining excellent agreement with the available data in the literature. By summing the elastic, electronically inelastic, and ionization cross sections, we estimated the total cross section. Our results were compared with previously reported theoretical and experimental data. For the elastic channel, the results obtained by considering the effects of multichannel coupling show good agreement with other calculations and with experimental data, including the position of the three π* shape resonances observed in the elastic channel. The inclusion of electronically excited states improved the agreement with the results obtained by Palihawadana et al.^16^ using the SMC method, highlighting the importance of multichannel coupling in the electron scattering process by molecules. The inelastic cross sections were grouped into specific sets of excited states, enabling comparison with experimental bands and other calculations. However, regarding electronic excitation, the overall agreement is still unsatisfactory. Significant differences were observed in both the shape and magnitude of the calculated curves when compared with available theoretical and experimental data. This discrepancy remains an open question. For the lower-energy bands, we found similarities in the behavior of our results, although they show many structures due to threshold effects. For the higher-energy bands, the discrepancies among the theoretical results become even more pronounced. This is partly due to the inadequate description of the electronically excited states—in these energy ranges, Rydberg states dominate, and these are not well represented within the approximation adopted in this work, which contributes to the observed discrepancies.

Pyrimidine is one of the simplest and most relevant molecular prototypes for investigating radiation-induced damage to nitrogenous bases of DNA and RNA. Due to its representative structure, it is widely used as both a theoretical and experimental model in studies of interactions between charged particles and biomolecules. In this work, we present a comprehensive set of cross sections—including elastic, electronic excitation, and ionization processes—which can serve as fundamental data for modeling and simulating particle interactions in biologically relevant molecular media. We believe that the results obtained here will significantly contribute to the scientific community, especially in the construction of reliable databases for studies on radiation-induced molecular damage.

However, we emphasize that a more accurate study of excitation cross sections is still required—a problem that remains an open question in the field of collision physics. More experiments and calculations are necessary to help in the understanding of the electronic-excitation/multichannel coupling problem. We hope that the results presented in this work will support and guide the scientific community in future investigations.
